# Supplementary material for: Thermal Traits of MNPs under High-Frequency Magnetic Fields: Disentangling the Effect of Size and Coating
Source: Nanomaterials (Basel). 2021 Mar 19;11(3):797. doi: 10.3390/nano11030797 (PMC8003606; doi:10.3390/nano11030797)
Supplement: Supplementary file 1 [file nanomaterials-11-00797-s001.pdf]

Supplementary Information for:
"Thermal traits of MNPs under high-frequency magnetic fields: disentangling the effect of size and coating."

D. Aurélio[1], J. Mikšátko[2], M. Veverka[1], M. Michlová[2], M. Kalbáč[2], J. Vejpravová[1]
[1] *Department of Condensed Matter Physics, Faculty of Mathematics and Physics, Charles University, Ke Karlovu 5, 121 16 Prague 2, Czech Republic.*
[2] *J. Heyrovsky Institute of Physical Chemistry of the Czech Academy of Sciences, v.v.i., Dolejškova 2155/3, 182 23 Prague 8, Czech Republic.*
(Dated: March 14, 2021)

DIFFUSE REFLECTANCE INFRARED FOURIER TRANSFORM SPECTROSCOPY.

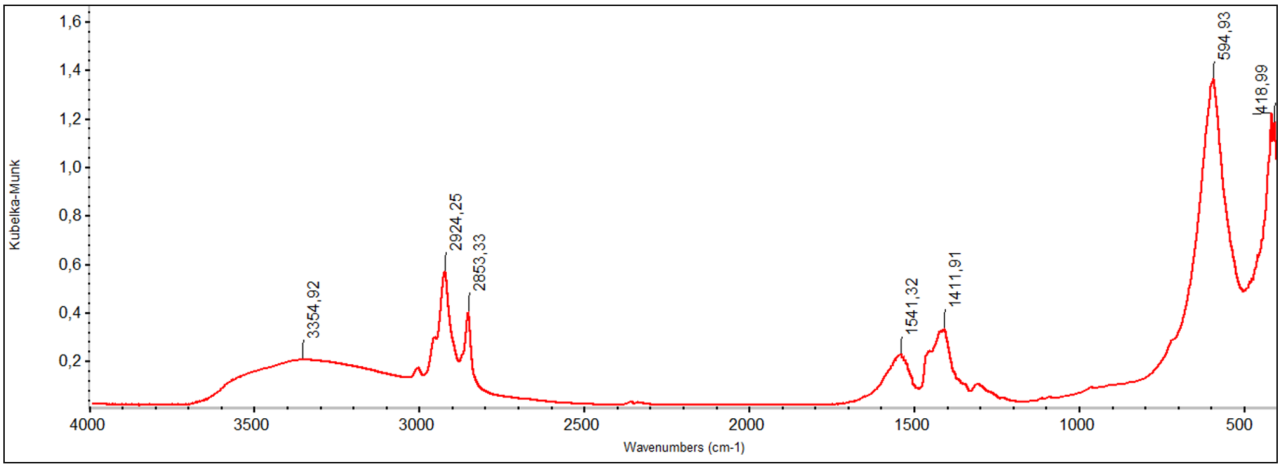

FIG. S1: DRIFTS spectrum of cobalt ferrites MNPs coated with OA in the mixture with KBr.

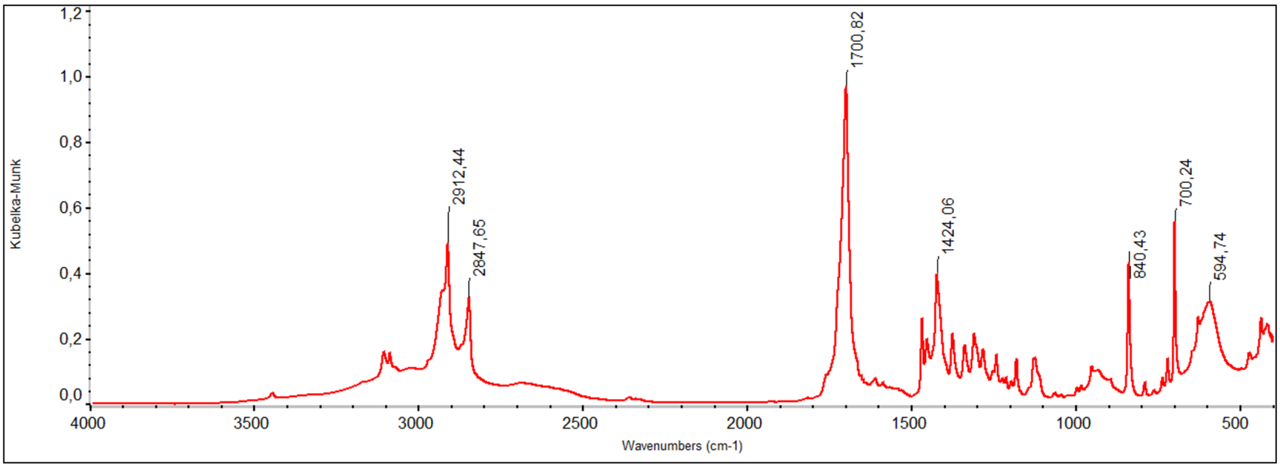

FIG. S2: DRIFTS spectrum of cobalt ferrite MNPs functionalized with MA (11-maleimidoundecanoic acid) with the band of C=O stretching at 1700 cm^{-1} together with two strong singlets of an out-of-plane bending of a maleimide C–H bond at 825 and 690 cm^{-1}, and two typical broad singlets at 600 and 420 cm^{-1} corresponding to the infrared absorption of spinel-structured $CoFe_2O_4$ (298 K, KBr, cm^{-1}).

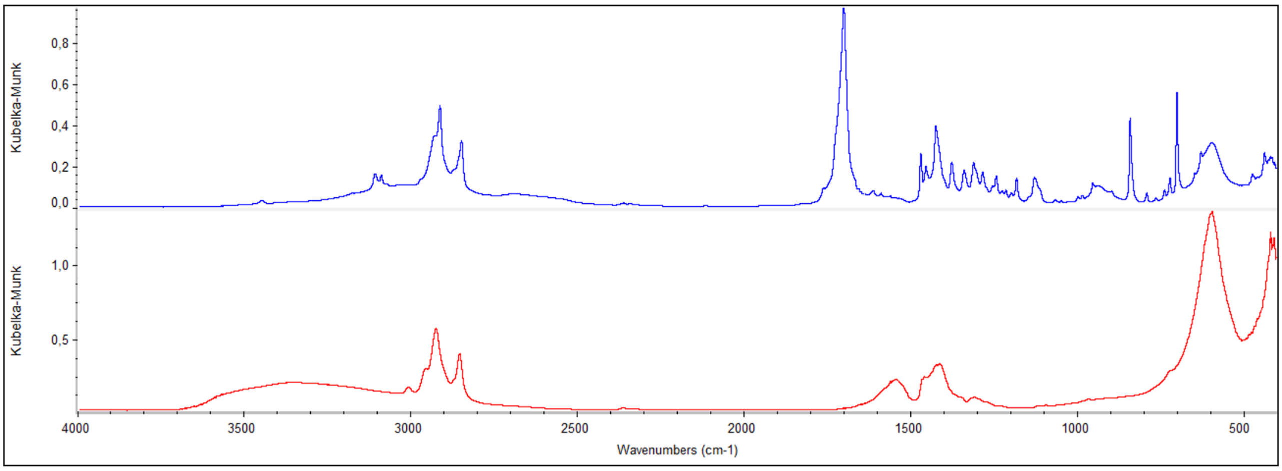

FIG. S3: Comparison of DRIFTS spectra of CoFe1_OA and CoFe functionalized with 11-maleimidoundecanoic acid (CoFe_MA).

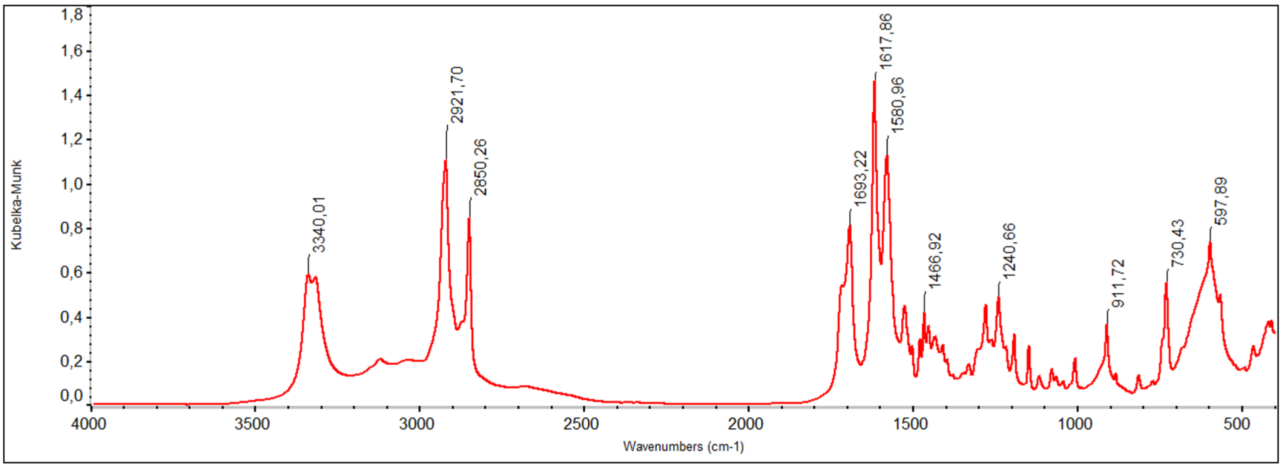

FIG. S4: DRIFTS spectrum of cobalt ferrites functionalized with 11-(furfurylureido)undecanoic acid with characteristic NH stretching bands around 3320 cm^{-1} together with C=O ureido stretching bands at around 1700 cm^{-1}. In addition, besides the clear presence of $–CH_2–$ stretching, the 2-substituted furan ring is confirmed by three bands at around 915-800 cm^{-1} originated from a C-H out-of-plane deformation. Two typical broad singlets corresponding to the infrared absorption of spinel-structured $CoFe_2O_4$ are present at 600 and 420 cm^{-1} (298 K, KBr, cm^{-1}).

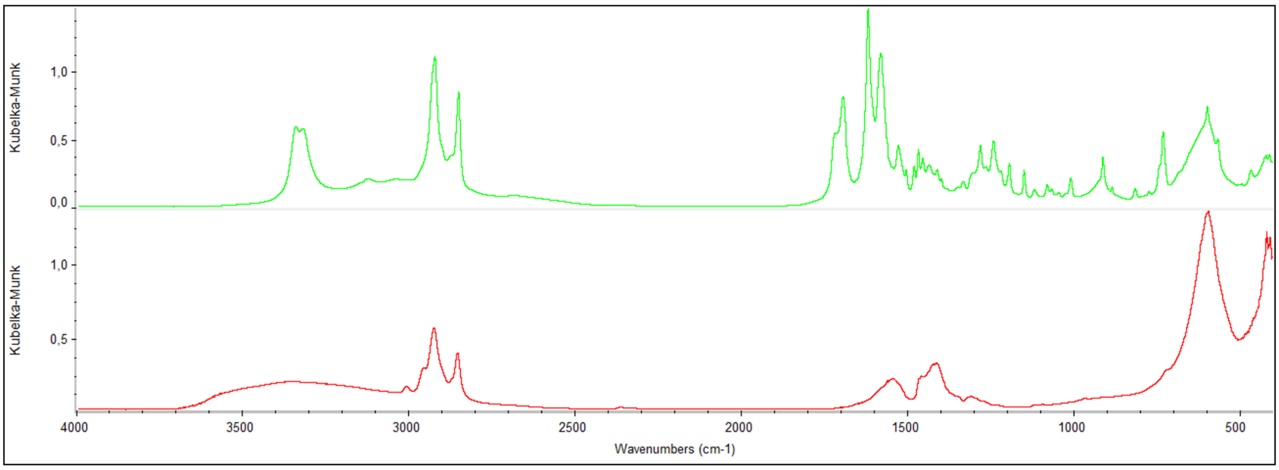

FIG. S5: Comparison of DRIFTS spectra of CoFe_OA and CoFe functionalized with 11-(furfurylureido)undecanoic acid (CoFe_FU).

SUMMARY ON HEATING MECHANISMS.

In small MNPs with the size below the single-domain limit, the heating mechanism is dominated by the MNP's relaxation losses. These are a combination of both Néel (τ_{N}) and Brownian (τ_{B}) relaxation mechanisms, which occur simultaneously (see Figure S6) and can be expressed as follows:[1–3].

$$\tau_{\mathrm{N}} = \tau_0 \mathrm{e}^{\frac{K V_{\mathrm{c}}}{k_{\mathrm{B}} T}} \text{ (s)} \qquad \tau_{\mathrm{B}} = \frac{3\eta V_{\mathrm{c}}}{k_{\mathrm{B}} T} \text{ (s)} \tag{1}$$

$$\frac{1}{\tau} = \frac{1}{\tau_{\mathrm{N}}} + \frac{1}{\tau_{\mathrm{B}}} \text{ (1/s)} \tag{2}$$

Where: K is the anisotropy constant, V_{c} the MNP volume, k_{B} the Boltzmann constant, T the temperature and τ_0 is the "event time" or "attempt frequency" (which typically; $\approx 10^{-9}$, 10^{-10} s), η is the viscosity of the surrounding medium and V_{h} the hydrodynamic volume.

Considering the general relaxation time given by the eqution 2, the heat is generated as a result of the MNP's macrospin alignment with the applied high-frequency MF, and subsequent relaxation back to its original direction. In the Néel mechanism, the physical position of the overall MNP (V_{h}) is kept fixed, while the superspin moment, μ rotates and aligns with the direction of the external field (Figure S6 (a)). Whereas in the Brownian mechanism the full MNP (V_{h}) rotates within the fluid medium so as to align with the external MF direction (Figure S6 (b)). Both regimes are expected to occur simultaneously and the dominant mechanism depends on the MNP and solvent properties, as well as on the MNP's concentration, temperature and parameters of the external MF.

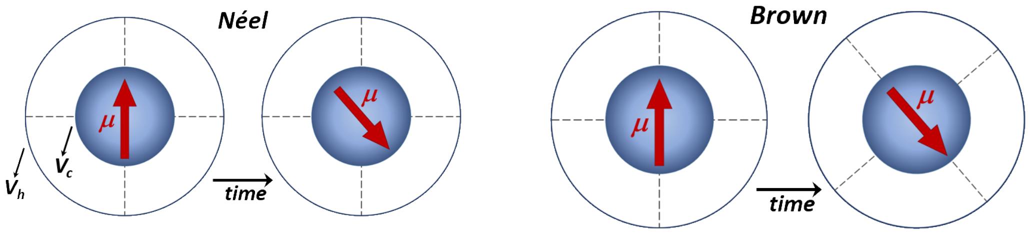

(a) Néel mechanism (b) Brownian mechanism

FIG. S6: Heating mechanisms. (a) Néel mechanism; where only the superspin moment μ of the core (V_{c}) rotates. (b) Brownian mechanism, where the entire MNP rotates within the fluid to align towards the external field.

DETAILS ON CALCULATION OF SELECTED MAGNETIC PARAMETERS.

The main analysis of the MNPs heating properties is done by determining their Specific Power Absorption (SPA). This quantity represents the capacity of a magnetic material to absorb energy from the high-frequency MF, and it is represented by the following equation; [2, 4]

$$\text{SPA} = \frac{m_{\text{NP}} c_{\text{NP}} + \text{m}_{\text{l}} \text{c}_{\text{l}}}{m_{\text{NP}}} \approx \frac{\delta_l c_{\text{l}}}{\varphi} \frac{\text{d}T}{\text{d}t} \qquad \text{(W/g)} \tag{3}$$

where: δ_l is the solvent density (g/ml); c_{l} the specific heat capacity of the solvent (J/g.K) and φ the NP concentration (g/ml). Note that the approximation shown in eq.3 is due to the mass on the MNP (m_{NP}) being much smaller than the mass of the solvent (m_{l}).

For the corrlation of the heating performance with the properties of the MNPs, we also derived other usefull magnetic parameters, such as the magnetic size (d_{MAG}). The d_{MAG} is directly related to the particle's magnetic moment and is determined by the following equation 4 [5];

$$d_{\text{MAG}} = 2^3 \sqrt{\frac{3}{4\pi} \frac{\mu_{\text{m}}}{\mu_{\text{uc}}} V_{\text{uc}}} \qquad \text{(m)} \tag{4}$$

where: d_{MAG} is the MNP diameter in the unit cell volume V_{uc} corresponding units; μ_{m} is the mean magnetic moment and μ_{uc} the unit cell magnetic moment, which for our $CoFe_2O_4$ samples is; 24 μ_{B} for inverse spinel and 56 μ_{B} for normal spinel.

The estimate of the effective anisotropy, for both normal and inverse spinel configuration, we used the following expression [6];

$$T_{\text{B}} = \frac{KV}{25 k_{\text{B}}} \qquad \text{(K)} \tag{5}$$

Considering that the MNPs in our samples are uniform and spherical, and that the magnetic properties arise mainly from the magnetically ordered part of the MNP represented by the d_{MAG}, we can assume the effective anisotropy in the form:

$$K_{\text{eff}} = \frac{6}{\pi} \frac{25 k_{\text{B}} T_{\text{B}}}{d_{\text{MAG}}^3} \qquad \text{(J/m}^3\text{)} \tag{6}$$

ADDITIONAL DATA ON THE CORRELATION OF HEATING PERFORMANCE AND MAGNETIC PARAMETERS.

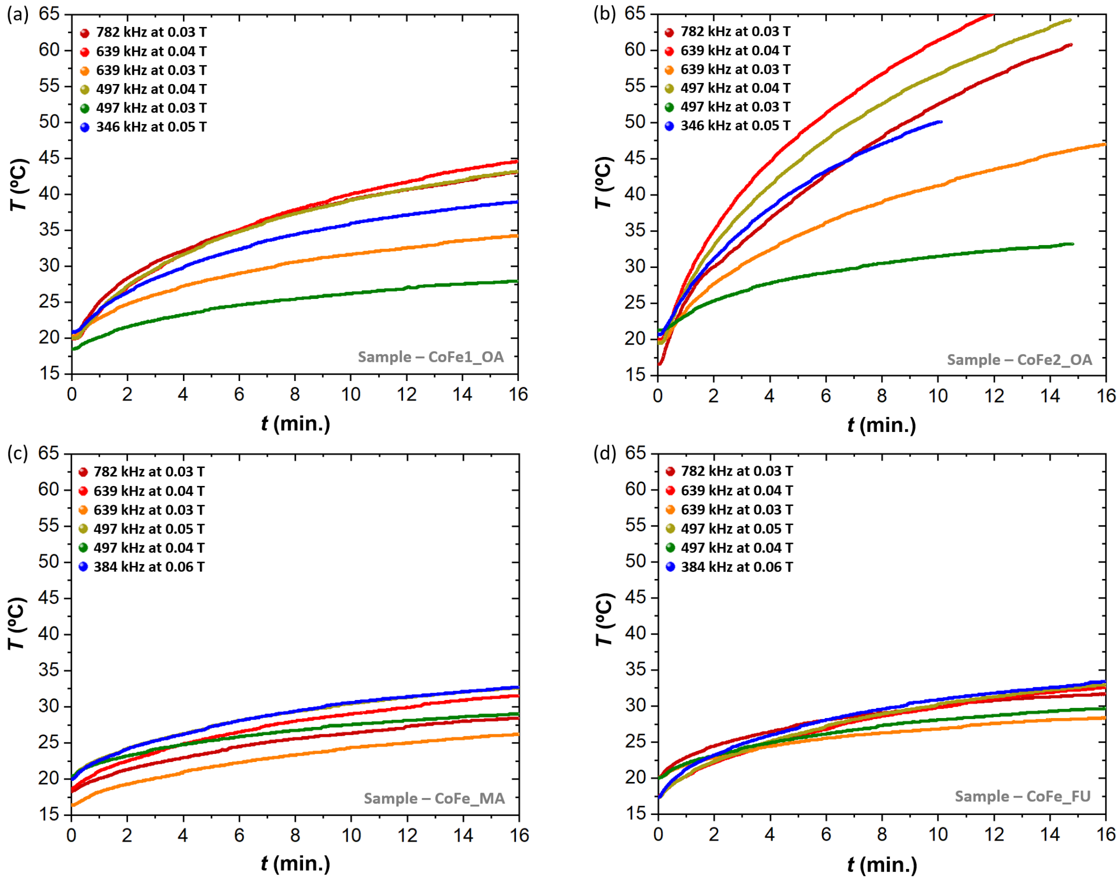

FIG. S7: Heating curves for different frequencies and amplitudes of the MF. (a) CoFe1_OA. (b) CoFe2_OA. (c) CoFe_MA. (d) CoFe_FU.

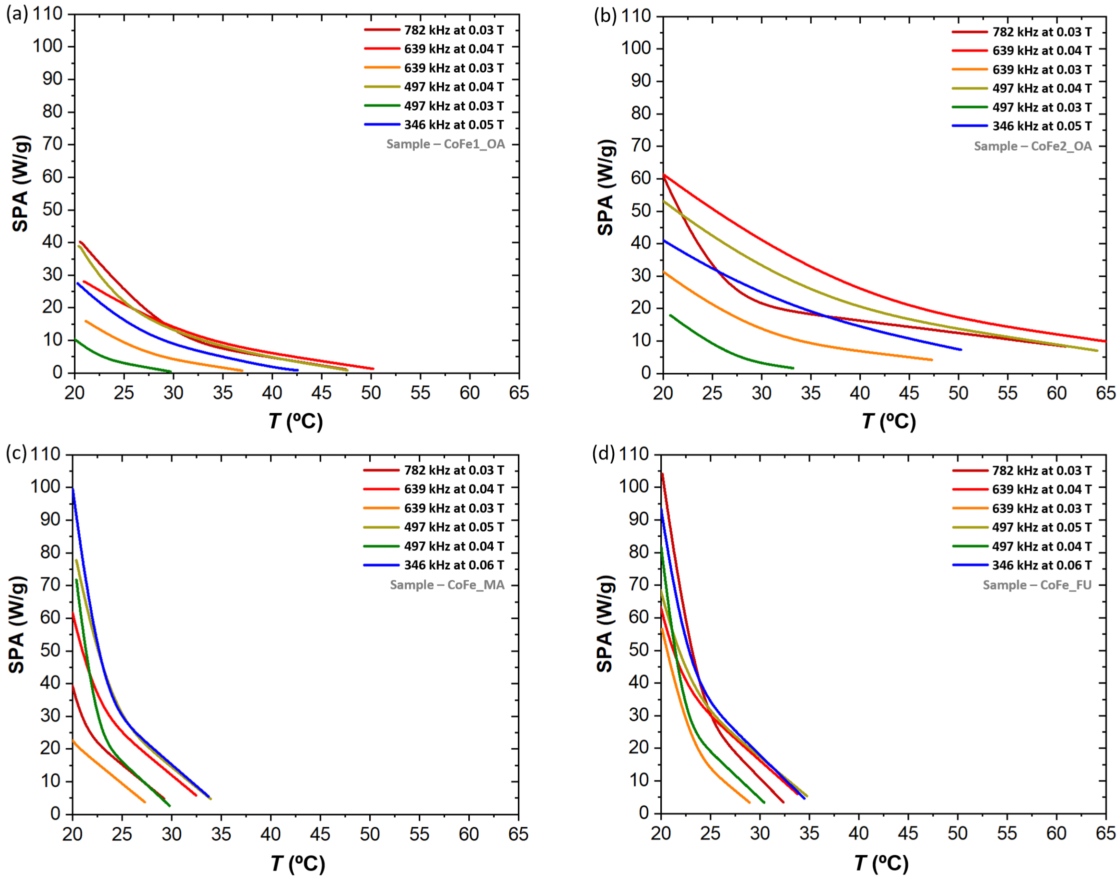

FIG. S8: SPA curves for different frequencies and amplitudes of the MF. (a) (a) CoFe1_OA. (b) CoFe2_OA. (c) CoFe_MA. (d) CoFe_FU.

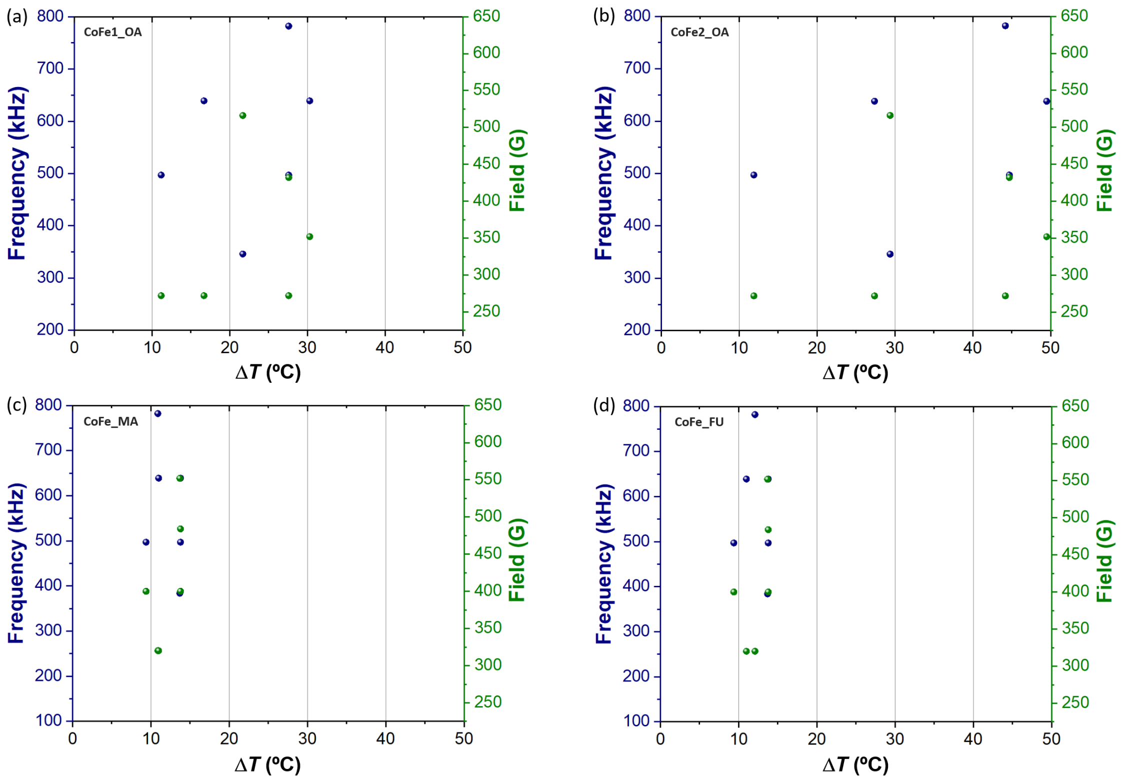

FIG. S9: Temperature changes (ΔT) at several frequencies and amplitudes of the MF. (a) CoFe1_OA. (b) CoFe2_OA. (c) CoFe_MA. (d) CoFe_FU.

[1] D. B. Reeves and J. B. Weaver, arXiv e-prints arXiv:1505.02450 (2015), 1505.02450.

[2] Z. Shaterabadi, G. Nabiyouni, and M. Soleymani, Progress in Biophysics and Molecular Biology **133**, 9 (2018), ISSN 0079-6107, URL http://www.sciencedirect.com/science/article/pii/S0079610717301499.

[3] T. E.Torres, E. Lima, M. P. Calatayud, B. Sanz, A. Ibarra, R. Fernández-Pacheco, A. Mayoral, C. Marquina, M. R. Ibarra, and F. G. Goya, Scientific Reports **9**, 3992 (2019), URL https://doi.org/10.1038/s41598-019-40341-y.

[4] M. Kallumadil, M. Tada, T. Nakagawa, M. Abe, P. Southern, and Q. A. Pankhurst, Journal of Magnetism and Magnetic Materials **321**, 1509 (2009), ISSN 0304-8853, proceedings of the Seventh International Conference on the Scientific and Clinical Applications of Magnetic Carriers, URL http://www.sciencedirect.com/science/article/pii/S0304885309001437.

[5] J. Mikšátko, D. Aurélio, P. Kovaříček, M. Michlová, M. Veverka, M. Fridrichová, I. Matulková, M. Žáček, M. Kalbáč, and J. Vejpravová, Nanoscale **11**, 16773 (2019), URL http://dx.doi.org/10.1039/C9NR03531A.

[6] G. Cullity and C. D. Graham, *Fine Particles and Thin Films* (John Wiley & Sons, Ltd, 2008), chap. 11, pp. 359–408, ISBN 9780470386323, https://onlinelibrary.wiley.com/doi/pdf/10.1002/9780470386323.ch11, URL https://onlinelibrary.wiley.com/doi/abs/10.1002/9780470386323.ch11.
